# The effect of adjuvant chemotherapy on survival in node negative colorectal cancer with or without perineural invasion: a systematic review and meta-analysis

**DOI:** 10.3389/fsurg.2023.1308757

**Published:** 2023-11-16

**Authors:** Hongan Ying, Jinfan Shao, Nansheng Liao, Xijuan Xu, Wenfeng Yu, Weiwen Hong

**Affiliations:** ^1^ Department of Geriatrics, Taizhou First People’s Hospital, Taizhou, China; ^2^ Department of Anus & Intestine Surgery, Taizhou First People’s Hospital, Taizhou, China; ^3^ Department of General Surgery, Taizhou First People’s Hospital, Taizhou, China

**Keywords:** perineural invasion, adjuvant chemotherapy, node negative, colorectal cancer, retrospective cohort

## Abstract

**Purpose:**

It was aimed at assessing the benefits of adjuvant chemotherapy (ACT) for patients with node-negative colorectal cancer (CRC) either with or without perineural invasion (PNI).

**Methods:**

We systematically searched PubMed, Cochrane Library, Embase, and Web of Science from database inception through October 1, 2023. Survival outcomes were analyzed using hazard ratios (HRs) and corresponding 95% confidence intervals (CIs). The methodological quality of included studies was assessed using the Newcastle-Ottawa Scale (NOS). Heterogeneity for the descriptive meta-analyses was quantified using the *I*^2^ statistic.

**Results:**

Ten studies included in this review. ACT improved overall survival (OS) (HR 0.52, 95% CI 0.40–0.69) and disease-free survival (DFS) (HR 0.53, 95% CI 0.35–0.82) in PNI + patients but did not affect DFS (HR 1.13, 95% CI 0.72–1.77) in PNI- patients. A disease-specific survival (DSS) benefit with chemotherapy was observed in PNI + (HR 0.76, 95% CI 0.58–0.99) and PNI- patients (HR 0.76, 95% CI 0.57–1.00). And PNI decreased DFS (HR 1.94, 95% CI 1.52–2.47) and OS (HR 1.75, 95% CI 0.96–3.17) in node-negative CRC.

**Conclusions:**

In conclusion, chemotherapy appears most beneficial for survival outcomes in node-negative patients with PNI, but may also confer some advantage in those without PNI.

**Systematic Review Registration:**

Identifier INPLASY2021120103.

## Introduction

Colorectal cancer is the third most common type of cancer in both men and women. Globally, almost 1.5 million new cases of CRC are diagnosed every year, of which more than a third are fatal (1). The most common cause of death is complications arising from metastasis (2). The primary treatment for stage I–II CRC is radical surgery (3). However, undetected micrometastases that persist after curative surgery may cause cancer recurrence (4). This micrometastasis is eradicated with ACT to enhance cure rates (5). It is unfortunate that few reliable prognostic and predictive markers exist to identify patients at a high risk for disease progression during the early stages of CRC (6). Stage II CRC recurrence rates range from 7.9%–22%, whereas only 2%–5% of patients benefit from ACT (7–10). Due to these reasons, the National Comprehensive Cancer Network (NCCN) does not recommend conventional ACT for stage II CRC unless certain risk factors exist. There were pT4 lesions, intestinal perforation, obstruction, 12-sample lymph nodes (LNs), lymph vascular invasion, PNI, poorly differentiated histology and margins that are positive, indeterminate, or close (11). Patients with these risk factors have a relatively poor prognosis (12). According to Lin et al., ACT was beneficial to patients with CRC and certain risk factors (13). In contrast, O'Connor et al. reported that ACT had no effect on any of these risk factors (14). Kumar et al. found that ACT was most effective for patients with pT4 in high-risk patients (15). Recent studies suggest, however, that ACT can benefit patients with PNI (16–18). PNI refers to tumor cells spreading through nerves. It was Bataskis who first described the prognostic value of PNI, which he defined as “tumor invasion around and through nerves (19).” PNI has been recognized as an unfavorable prognostic factor in CRC since it is associated with poor survival rates (20).

ACT, however, remains controversial because it is unclear whether these patients will benefit patients with PNI (21). This study was conducted to determine whether node-negative CRC patients with and without PNI receive different benefits from ACT.

## Materials and methods

### Search strategy

Our search focused on academic papers published in English between inception through October 1, 2023 in PubMed, Cochrane Library, Web of Science, and Embase databases. The following keywords were used: “perineural invasion”, “PNI”, “colon cancer”, “rectal neoplasms”, “Corectal cancer”, “colorectal neoplasms”, “adjuvant chemotherapy”, “cohort”, “randomized controlled trial”, and “randomized trial”. Additionally, we searched the references of relevant articles.

### Selection criteria and exclusion criteria

Studies were included if they met the following criteria: (1) Enrolled patients with stage II CRC who underwent radical resection, confirmed by postoperative histopathology. (2) Assessed the association between PNI and survival among patients receiving ACT. (3) Published in English. (4) Reported sufficient data to calculate HRs and 95% CIs. Studies were excluded if they: (1) Were not published in English. (2) Included node-positive or mixed stage CRC patients. (3) Were case reports or case series with <50 patients. (4) Did not report outcomes of interest including OS, DFS.

### Data extraction and quality assessment

The researchers (W. Yu and H. Ying) independently assessed the eligibility of all the studies and extracted the following information: The first author's name, the country in which the study was conducted, the sample size, the year of the study, the ages of the participants, the stage of their cancer, the chemotherapy regimen, and the period of follow-up. As well as OS, DFS, DSS, recurrence-free survival (RFS), and NOS. We consulted with a third reviewer (W. Hong) to resolve any discrepancies between the reviewers. In order to rate the quality of the articles, we used the NOS score. Articles that have an NOS score >6 (on a scale of 0–9) were considered to be of high quality (22).

### Risk of bias analysis

Using non-parametric correlation tests, we examined the association between quality of reporting and HR. Begg and Egger tests were also conducted to determine whether publication bias was present (23).

### Statistical analysis

Our analysis used HRs and 95% CIs to compare PNI with survival. When HRs and 95% CIs were not included, data were derived from survival curves according to Parmar et al. and Tierney et al. (24, 25). Study heterogeneity was examined using *I*^2^ statistics. Whenever there was obvious heterogeneity, as indicated by a *p*-value < 0.10 or *I*^2^ exceeding 50%, a random effect model was used. In other cases, a fixed effect model was used. Our findings were further enhanced by performing meta-regressions and subgroup analyses in order to identify the sources of heterogeneity. We conducted sensitivity analysis to determine the stability of our combined results, and we assessed publication bias using the Begg and Egger test (26, 27). Statistical significance was set at *p* < 0.05 using STATA 16.0 (Stata Corporation, College Station, TX, USA).

## Results

### Search results and quality assessment

**Figure 1:**
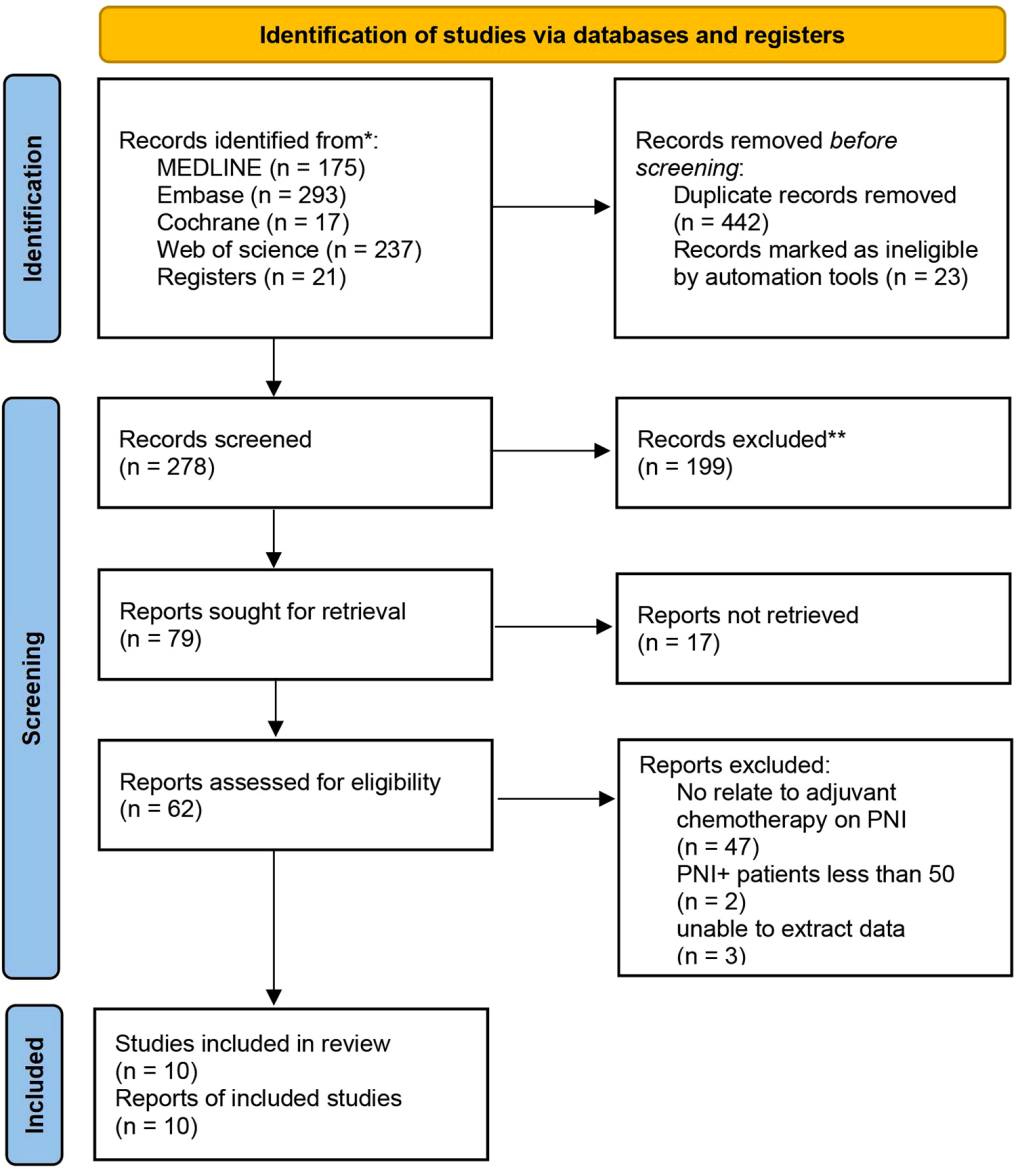
Flow chart of patient search strategy.

We conducted electronic searches of MEDLINE, Embase, Cochrane Library, and Web of Science, which yielded 743 studies. An additional 21 studies were identified from reference lists. After removing 442 duplicate records, 322 studies underwent title and abstract screening, of which 199 were excluded as Records excluded. The full texts of the remaining 123 studies were assessed; 17 studies could not be retrieved and 52 further studies were excluded based on predefined criteria. Ultimately, 10 studies met the inclusion criteria and were included in the systematic review and meta-analysis, comprising data on 118,529 patients in total. The study selection process is outlined in the PRISMA flow diagram (Figure 1). All patients underwent curative-intent resection of their CRC prior to ACT. Some studies also analyzed the high-risk factors after colon cancer surgery. Table 1 summarizes the 10 retrospective cohort studies included in the systematic review. These studies involved 118,529 patients with stage II CRC who underwent surgery. The studies compared ACT vs. no chemotherapy and reported on outcomes including OS, DFS, and recurrence. Follow-up times ranged from 5 to 10 years. We assessed the quality of ten articles by using the NOS score since they were retrospective cohort studies. A total of seven articles scored 7 points and six articles scored 8 points, with the main loss being the study controls for confounding factors (Table 2).

**Table 1:** Characteristics of included studies.

References	Country	Age (mean)	Timeframe	Stage	Tumor location	Patients	PNI + patients (ACT)	Study design	Follow-up	Outcome	NOS score
Skancke	USA	70	2010–2014	II	Colon cancer	32,493	2,404 (480)	RSC	5.0	OS	8
Mirkin	USA	72	2004–2012	I, II	Colon cancer	21,488	987 (202)	RSC	5.0	OS	8
Huh	Korea	63	2001–2006	II	Colorectal	341	57 (46)	RSC	5.0	DFS	7
Cienfuegos	Spain	64	2000–2012	I, II	Colon cancer	507	57 (36)	RSC	10	DFS	7
Tu	China	65	2010–2015	II	Colon cancer	57,255	2,372 (486)	RSC	7.0	DSS	8
Kumar	Canada	67	1999–2008	II	Colon cancer	1,697	89 (37)	RSC	5.3	OS,DSS,RFS	8
Loree	British	69	1999–2009	II	Rectal cancer	851	51 (18)	RSC	8.5	DFS, RFS	7
Song	Korea	62	2005–2014	II	Rectal cancer	1,232	185 (150)	RSC	5.0	RFS, DFS	8
Babcock	USA	NR	2010–2013	II	Colon cancer	2,374	248 (52)	RSC	5.0	OS	8
Morris	Germany	65	1993–2003	II	Colon cancer	812	47 (7)	RSC	5.0	OS	7

ACT, adjuvant chemotherapy; PNI+, perineural invasion positive; RSC, retrospective cohort; OS, overall survival; DFS, disease-free survival; NOS, Newcastle-Ottawa scale; NR, not reported.

**Table 2:** Results of quality assessment using the Newcastle-Ottawa scale.

				Selection		Comparability		Outcome			
References	Years	REC	SNEC	AE	DO	SC	AF	AO	FU	AFU	Total score
Skancke	2019	1	1	1	1	1	0	1	1	1	8
Mirkin	2017	1	1	1	1	1	0	1	1	1	8
Huh	2010	1	1	1	0	1	0	1	1	1	7
Cienfuegos	2017	1	1	1	1	1	0	1	1	1	7
Tu	2021	1	1	1	1	1	0	1	1	1	8
Kumar	2015	1	1	1	1	1	0	1	1	1	8
Jonathan	2016	1	1	1	0	1	0	1	1	1	7
Song	2019	1	1	1	1	1	0	1	1	1	8
Babcock	2018	1	1	1	1	1	0	1	1	1	8
Morris	2007	1	1	1	0	1	0	1	1	1	7

AE, indicates ascertainment of exposure; AF, study controls for any additional factors; AFU, adequacy of follow-up of cohorts; ″1″ means that the study is satisfied the item and “0″ means the opposite situation. AO, assessment of outcome; DO, demonstration that outcome of interest was not present at start of study; FU, follow-up long enough for outcomes to occur; REC, representativeness of the exposed cohort; SC, study controls for age; SNEC, selection of the nonexposed cohort.

### Effect of adjuvant chemotherapy on perineural invasion

We evaluated the survival of node-negative CRC patients who received ACT compared to no chemotherapy. Across the 10 included studies, 6,196 patients had PNI, with 1,467 receiving ACT. The prevalence of PNI ranged from 5.2% to 11.3% based on tumor location. OS was analyzed in 6 studies comprising 3,794 PNI + patients, of whom 786 underwent ACT, as well as 54,177 PNI- patients, including 6,535 who received ACT (12, 15, 28–31). DFS was examined in 4 studies including 344 PNI + patients (with 62 receiving ACT) and 3,285 PNI- patients (with 1,191 receiving ACT) (29, 32–34). Two studies with 2,461 PNI + (262 ACT) and 55,257 PNI- (4,675 ACT) patients analyzed DSS (15, 35). Recurrence-free survival (RFS) was assessed in 2 studies: one with 108 PNI + patients (43 ACT) and another with 2,498 PNI- patients (471 ACT) (15, 29). RFS was improved with ACT in PNI + patients (HR 0.79, 95% CI 0.42–1.46, *I*^2^ = 0%), but RFS data were unavailable for PNI- patients (Table 1).

**Figure 2:**
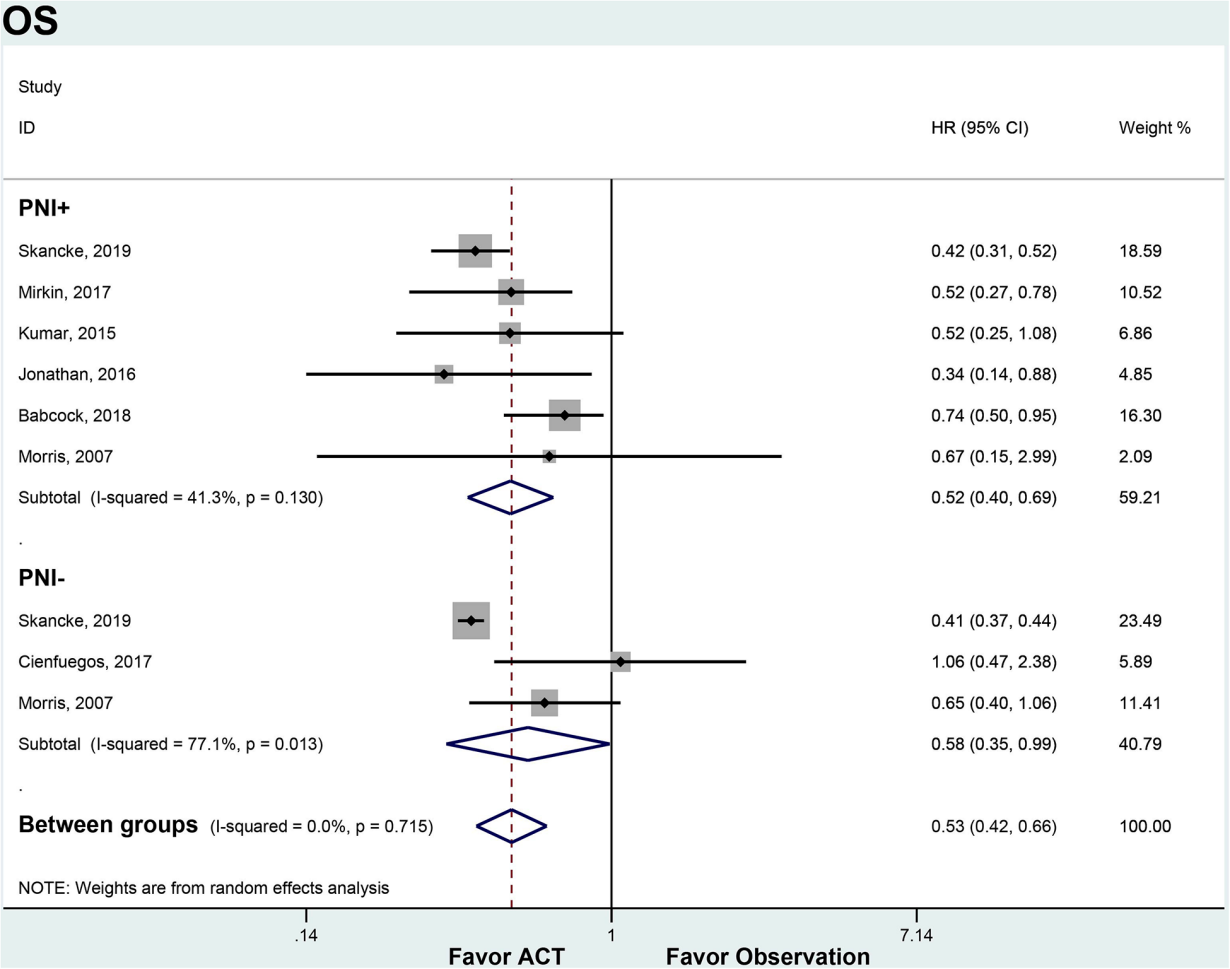
ACT versus observation-only patients stratified by PNI, OS. Diamond represents the pooled effect estimate of the overall analysis. Data are represented as HRs with 95% CIs. Inter-study heterogeneity quantified by *I*^2^ with significance *p* < 0.10. HR, hazard ratio; OS, overall survival; ACT, adjuvant chemotherapy; PNI, perineural invasion.

We compared OS, DFS and DSS between patients who received ACT and those who observation only, stratified by PNI status. For patients with node-negative CRC and PNI+, ACT was associated with significantly improved OS compared to observation (HR 0.52, 95% CI 0.40–0.69). There was moderate heterogeneity between the 3 included studies (*I*^2 ^= 41.3%, *p* = 0.130). In the PNI- subgroup, ACT also conferred an OS benefit over observation (HR 0.52, 95% CI 0.27–0.78). However, there was substantial heterogeneity between the 2 studies in this analysis (*I*^2 ^= 77.1%, *p* = 0.013). ACT appeared to improve OS regardless of PNI status. The OS benefit with ACT was similar between PNI + and PNI- patients (Figure 2).

**Figure 3:**
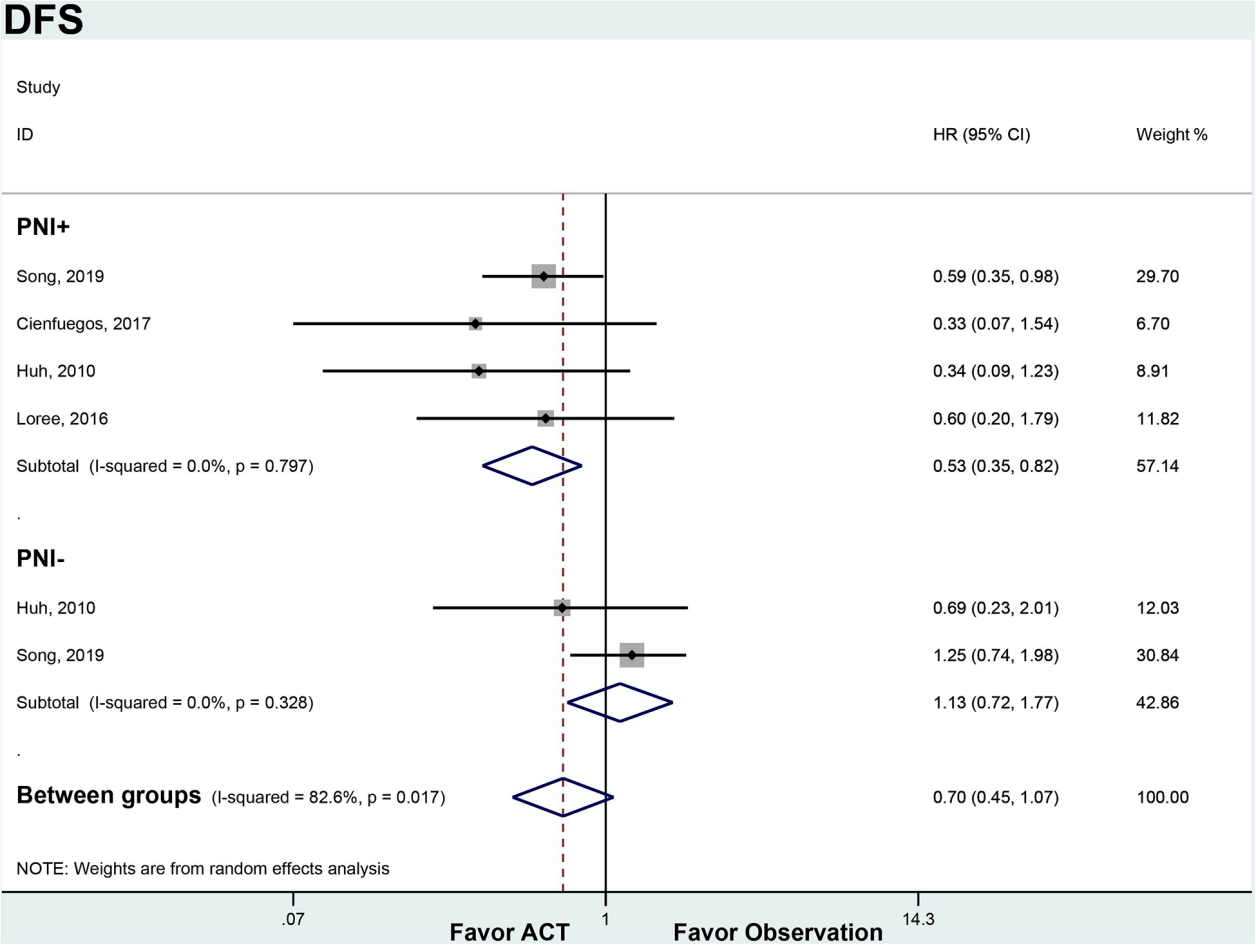
ACT versus observation-only patients stratified by PNI, DFS. Diamond represents the pooled effect estimate of the overall analysis. Data are represented as HRs with 95% CIs. Inter-study heterogeneity quantified by *I*^2^ with significance *p* < 0.10. HR, hazard ratio; DFS, disease-free survival; ACT, adjuvant chemotherapy; PNI, perineural invasion.

Among PNI + patients, ACT significantly improved DFS compared to observation alone (HR 0.53, 95% CI 0.35–0.82). There was no heterogeneity between the 4 studies in this subgroup (*I*^2 ^= 0%, *p* = 0.797). In the PNI- subgroup, ACT did not provide a DFS benefit over observation (HR 1.13, 95% CI 0.72–1.77). No significant heterogeneity was found between the 2 PNI- studies (*I*^2 ^= 0%, *p* = 0.328). ACT appeared to improve DFS in node-negative CRC patients with PNI, but not in those without PNI (Figure 3).

**Figure 4:**
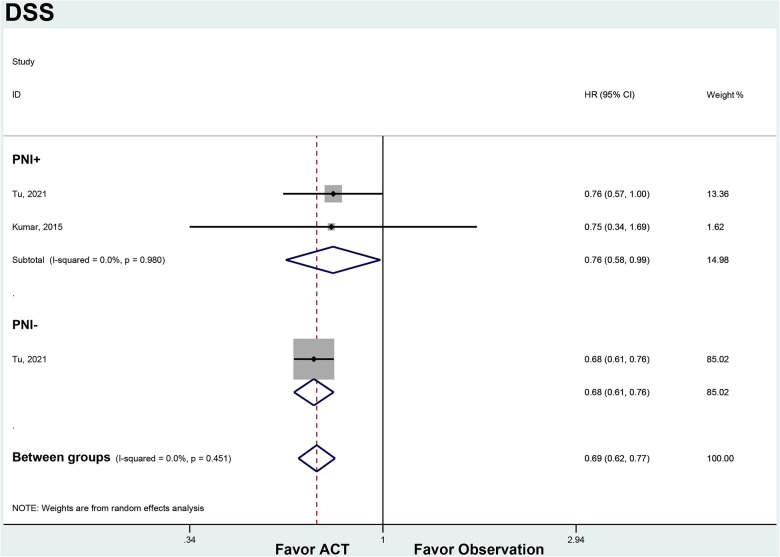
ACT versus observation-only patients stratified by PNI, DFS. Diamond represents the pooled effect estimate of the overall analysis. Data are represented as HRs with 95% CIs. Inter-study heterogeneity quantified by *I*^2^ with significance *p* < 0.10. HR, hazard ratio; DSS, disease-specific survival; ACT, adjuvant chemotherapy; PNI, perineural invasion.

In the PNI + subgroup, ACT was associated with improved DSS compared to observation (HR 0.76, 95% CI 0.58–0.99). There was no heterogeneity between the 2 studies (*I*^2 ^= 0%, *p* = 0.980). For PNI- patients, ACT also showed a trend towards improved DSS over observation that did not reach statistical significance (HR 0.76, 95% CI 0.57–1.00). Only 1 study was available for this subgroup analysis (Figure 4).

### Effect of perineural invasion on survival

**Figure 5:**
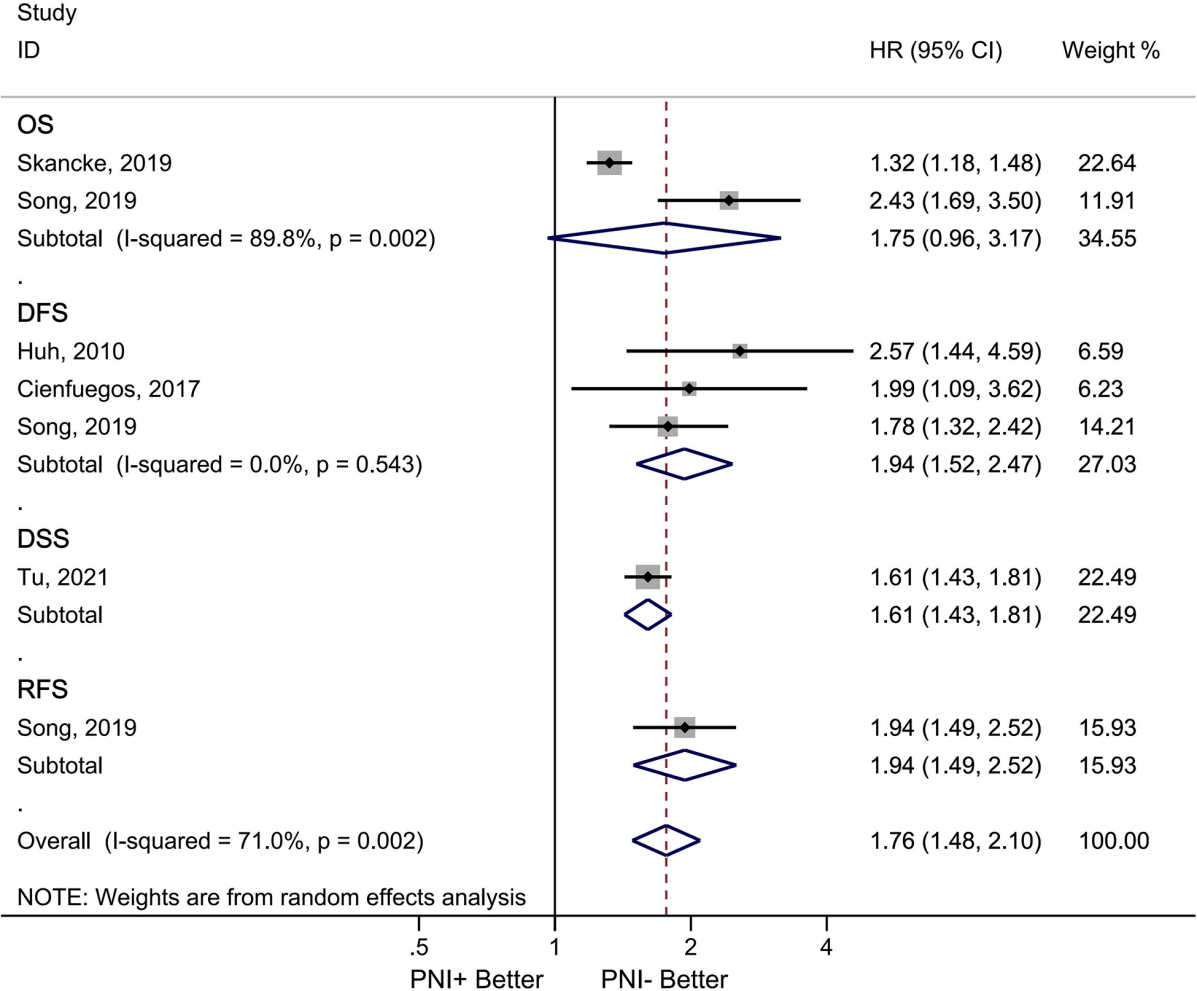
Association between PNI and survival in node negative colorectal cancer patients. Diamond represents the pooled effect estimate of the overall analysis. Data are represented as HRs with 95% CIs. Inter-study heterogeneity quantified by *I*^2^ with significance *p* < 0.10, HR, hazard ratio; ACT, adjuvant chemotherapy; PNI, perineural invasion; OS, overall survival; DFS, disease free survival overal; DSS, disease-specific survival; RFS, recurrence-free survival; PNI, perineural invasion.

Five studies involving 91,828 patients provided data on the impact of PNI on survival (28, 32–35). In three studies, PNI was found to decrease DFS (HR = 1.94, 95% CI = 1.52–2.47, *p* < 0.001). There was no significant heterogeneity between studies (*I*^2^ = 0.00%, *p* < 0.001). There were two studies analyzing the OS (28, 34). The OS decreased in the presence of PNI (HR = 1.75, 95% CI = 0.96–3.17). Significant heterogeneity was observed between studies (*I*^2^ = 89.8%, *p* = 0.002) (Figure 5).

### Sensitivity analysis

Fixed effects and random effects models were compared to analyze prognosis (OS) in patients with PNI who were treated with ACT.

**Figure 6:**
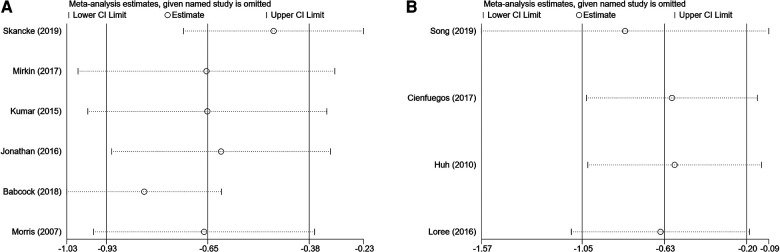
Sensitivity analysis of overall high-risk factors receiving adjuvant hemotherapy on OS (**A**) and disease-free survival (**B**).

We analyzed the prognosis (OS) of patients with PNI who received ACT by comparing fixed effect and random effect models. OS did not differ significantly between the two models (fixed effect model: HR = 0.51, 95% CI = 0.43–0.61, random effect model: HR = 0.52, 95% CI = 0.40–0.69). In the sensitivity analysis, we arbitrarily deleted the OS and DFS literature, which did not affect the results of this study (Figure 6).

### Publication bias

Our analysis included ten studies, but the subgroup studies were relatively few because they assessed different outcomes. There is an inherent risk of public bias in all reviews. According to Egger and Begg tests (Egger test: *p* = 0.189; Begg test: *p* = 0.308), DFS analysis did not detect a significant publication bias. In addition, the DFS analysis found no evidence of publication bias (Egger test: *p* = 0.925; Begg's test: *p* = 1.00).

## Discussion

This systematic review and meta-analysis examined the efficacy of ACT for node-negative CRC stratified by PNI status. Our results suggest that chemotherapy improves overall and DFS in patients with PNI, but may not affect DFS in those without PNI.

OS was significantly improved with ACT vs. observation in the PNI + subgroup (HR 0.52, 95% CI 0.40–0.69), consistent with prior studies showing a survival benefit for high-risk stage II patients receiving chemotherapy (36, 37). A recent cohort study of 500 colon cancer patients also found the addition of oxaliplatin to standard 5-FU chemotherapy prolonged OS and DFS selectively in the subgroup with PNI (33). The survival gain seen with chemotherapy in PNI + patients may be due to eradication of occult micrometastases not detectable on standard pathology (38). Interestingly, we also observed an OS benefit with chemotherapy in the PNI- subgroup (HR 0.52, 95% CI 0.27–0.78), although prior analyses have been conflicting (10, 39). The reason for improved OS with chemotherapy even for lower risk PNI- patients is unclear and warrants investigation.

DFS was significantly improved by chemotherapy in the PNI + subgroup (HR 0.53, 95% CI 0.35–0.82) but not in the PNI- subgroup (HR 1.13, 95% CI 0.72–1.77). These findings align with other studies demonstrating PNI is an independent prognostic factor for DFS (40). A potential explanation is that PNI + tumors are more aggressive and prone to early micrometastases or local recurrence after surgery that is eradicated by chemotherapy (41). The lack of DFS benefit with chemotherapy in PNI- patients highlights the need for risk-stratified treatment approaches to avoid over-treatment (42). Recent data suggest molecular profiling may help further stratify risk in node negative CRC (43).

This study has several limitations. The pooled sample size was relatively small for PNI subgroup analyses, particularly for secondary outcomes like DFS and DSS, warranting cautious interpretation. Publication bias remains a concern given the limited number of studies. There was heterogeneity between studies that may relate to differences in chemotherapy regimens, follow-up times, and underlying study populations. The retrospective observational nature of the included studies also has inherent biases compared to prospective trials. And this systematic review included studies published over a long timespan, ranging from 1993 to 2015. The inclusion of literature covering many decades could introduce bias, as changes in cancer treatments, staging modalities, and other factors over time may impact outcomes. Despite these limitations, this systematic review provides a comprehensive synthesis of current evidence regarding efficacy of ACT in early stage CRC with vs. without PNI.

ACT appears to improve survival outcomes primarily in node-negative CRC patients with PNI. PNI may be an important factor to guide chemotherapy decisions in this population. Additional well-designed prospective studies are needed to clarify the risk-benefit ratio of adjuvant treatment based on PNI status. Future research should also examine how emerging prognostic factors and individualized risk prediction models can optimize personalized adjuvant therapy for early stage CRC.

## Conclusion

ACT improved OS and DSS in node-negative CRC patients regardless of PNI status. But DFS benefit with chemotherapy was observed only in patients with PNI. Overall, chemotherapy appears most beneficial for survival outcomes in node-negative patients with PNI, but may also confer some advantage in those without invasion.

## Data Availability

The original contributions presented in the study are included in the article/Supplementary Materials, further inquiries can be directed to the corresponding author.
